# Simulation and Mitigation of the Wrap-Around Artifact in the MRI Image

**DOI:** 10.3389/fncom.2021.746549

**Published:** 2021-10-21

**Authors:** Runze Hu, Rui Yang, Yutao Liu, Xiu Li

**Affiliations:** ^1^Department of Information Science and Technology, Tsinghua Shenzhen International Graduate School, Tsinghua University, Shenzhen, China; ^2^School of Computer Science and Technology, Ocean University of China, Qingdao, China

**Keywords:** magnetic resonance imaging (MRI), wrap-around artifact, deep learning, image quality (IQ), image restoration

## Abstract

Magnetic resonance imaging (MRI) is an essential clinical imaging modality for diagnosis and medical research, while various artifacts occur during the acquisition of MRI image, resulting in severe degradation of the perceptual quality and diagnostic efficacy. To tackle such challenges, this study deals with one of the most frequent artifact sources, namely the wrap-around artifact. In particular, given that the MRI data are limited and difficult to access, we first propose a method to simulate the wrap-around artifact on the artifact-free MRI image to increase the quantity of MRI data. Then, an image restoration technique, based on the deep neural networks, is proposed for wrap-around artifact reduction and overall perceptual quality improvement. This study presents a comprehensive analysis regarding both the occurrence of and reduction in the wrap-around artifact, with the aim of facilitating the detection and mitigation of MRI artifacts in clinical situations.

## 1. Introduction

Magnetic resonance imaging (MRI) has become one of the most essential means in disease diagnostics and management. It deepens our understanding of the pathology involved in the development and progression of the disease. An MRI image is generally constructed using the Fourier transform (FT) method. The MRI signal is obtained by the interaction between the hydrogen atoms and the external electromagnetic fields. This signal is then encoded into the phase information and frequency information that are subsequently utilized to construct the spatial frequency map, also known as the K-space. The inverse Fourier transform (iFT) can be used to reconstruct the K-space data into the human-interpretable image (Gallagher et al., 2008). Although the MRI technique possesses numerous merits in clinical trials, such as radiation-free and high-contrast imaging, artifacts occur throughout the entire image acquisition process, from the MRI signal generation to the image display, which can significantly deteriorate the perceptual quality of the MRI image and subsequently affect the reliability of diagnosis (Bellon et al., 1986; Liu et al., 2018, 2019, 2020b; Zhai et al., 2020). Thus, it is crucial to effectively detect and eliminate artifacts of MRI image. This study hereby deals with one of the most common artifacts of MRI, namely the wrap-around artifact (also known as the aliasing artifact). We propose a novel artifact reduction framework to reduce the wrap-around artifact of the MRI image while improving the image perceptual quality.

Wrap-around artifact occurs when the scanned area of the human body exceeds the predefined field of view (FOV). These areas outside the FOV cannot be properly encoded relative to their actual position and are wrapped back into the opposite side of the image, resulting in the wrapped information reappearing on the other side of the image and subsequently cannot be distinguished from the objects inside the FOV. The wrap-around artifact can be further classified into frequency-related and phase-related. During the imaging process, there are a number of classical ways to mitigate the wrap-around artifact (Chen et al., 2013). The frequency-related artifact can be mitigated by the oversampling scheme that increases the density of the K-space frequency data and thus increases the FOV. As for the wrap-around in the phase-encode direction, we can swap phase and frequency directions such that the phase direction is oriented in the smallest direction. This method is straightforward while maintains the same spatial resolution. However, it may induce other artifacts to the MRI image, i.e., chemical shift artifact. Another method for reducing the phase-related artifact is to double the FOV in the phase direction, yet it may lower the spatial resolution. These remedies are only operational during the process of MR imaging. However, radiologists generally face post-operated (reconstructed) MRI image without knowing the occurrence of the artifact in the imaging processing. Eliminating the wrap-around artifact from the post-operated MRI image has remained a major deterrent to clinical adoption.

Numerous efforts for MRI artifacts reduction have been made in the last decades. Yang et al. (2001) proposed a maximum likelihood-based method to remove the ringing artifact, in which the prior knowledge of MRI, i.e., the sampled low-frequency data points, was adopted to deduce the high-frequency data in the K-space. This method aims to increase the high-frequency information and thus alleviate the artifact. Lee (1998) designed a Bayesian framework with the regularization scheme to reduce the MRI artifacts. This framework deduces the posterior probability of the output image by the likelihood of sampled spatial information and the local spatial structure of the input image. Yatchenko et al. (2013) mitigated the ringing artifact by computing the average edge-normal and edge tangential derivatives in the edge area of the image. In Guo and Huang (2009), a k-means-based method was proposed to remove the MRI artifact. The maximum likelihood method was at first employed to detect the artifact of the image. Then, the detected structures were fitted to a k-means model to map the neighboring pixel values and the estimation region. Sebastiani and Barone (1995) proposed to use the Markov random field to model the errors arisen in the truncation and characteristics of the Fourier series. The modeled errors can be utilized to implement artifact removal.

In addition to these model-based approaches, recent years have seen the prosperity of the deep learning-based techniques for the MRI artifact reduction. Lee et al. (2017) proposed a multi-scale deep neural network to remove the wrap-around artifact. This neural network estimates the area of a wrap-around artifact based on the distorted magnitude and the phase information of the input image. The removal of wrap-around artifact can be achieved by subtracting the estimated artifact area from the input image. Yang et al. (2017) proposed a de-aliasing strategy based on the conditional generative adversarial networks. The adversarial loss of this model incorporates three typical losses, i.e., the pixel-wise loss, frequency information loss, and perceptual loss, in order to better learn the texture and edge information, thereby improving the quality of the MRI image reconstructed from undersampled k-space data. The work in Hyun et al. (2018) presented a deep learning-based sample strategy to reconstruct the MR image from the undersampled k-space data while enhancing the image quality. This strategy adopts the uniform sampling method to obtain phase information of the image so that the details of the corrupted area of the image are preserved after Fourier transform. Consequently, the deep learning model can effectively learn the features of the wrap-around artifact.

Although the abovementioned model and learning-based methods have shown great potential in reducing the MRI artifacts, their capability for clinical practice is restricted. The reason is fourfold. First, many methods, i.e., Yang et al. (2001) and Guo and Huang (2009), directly manipulate the k-space data, which could inadvertently remove the non-artifact information, such as the anatomical or pathological details. Second, some deep learning methods, i.e., Yang et al. (2017), are based on the generative adversarial network (GAN), where the MRI image is synthesized from the given samples. This strategy is not very reliable since the synthesized MRI data may contain fake information, which can complicate the pathologic diagnosis. Third, in the context of Bayesian framework, such as Lee (1998) and Sebastiani and Barone (1995), reconstructing the MRI image from the undersampled k-space data is practically an ill-posed problem, and the rate of convergence of these methods remains questionable. Finally, one of the main limitations of the learning-based method is the scarcity of MRI data. Nevertheless, given the sensitivity and confidentiality of clinical data, it is rather difficult to obtain adequate MRI data, which severely restricts the development of learning-based methods.

We herein propose a novel wrap-around artifact reduction framework to address the aforementioned issues. The proposed framework comprises two stages, namely, artifact simulation and image enhancement. For the artifact simulation, we design an artifact occurrence mechanism to simulate the characteristics of the wrap-around artifact. Two parameters are designed to describe the characteristics of the wrap-around artifact. The first parameter determines the size of the wrapped area indicating how much area of the MRI image is corrupted by the artifact. The second parameter describes the intensity of the wrapped area, which is closely related to the distortion level of the MRI image. A large intensity may completely contaminate the wrapped area, of the MRI image, resulting in the difficulty of artifact removal. These two parameters work jointly to simulate the wrap-around artifact.

For the image enhancement, we propose a deep neural network (DNN) to remove the wrap-around artifact while improving the overall perceptual quality of the MRI image (Min et al., 2020a,b). The proposed DNN is based on the U-net network owing to its powerful performance in medical image processing. The DNN composes of two phases, i.e., artifact estimation and deep elimination. In the artifact estimation, a U-net-based network is trained by pairing artifacted MRI images with the corresponding artifact patterns. This enables the network to accurately estimate the wrapped area of the artifacted MRI image, which can be subsequently utilized to assist the network training at the second phase of deep elimination. As for the deep elimination, an end-to-end U-net based network is built, in which the inputs are the artifacted MRI images and the outputs are the artifact-free MRI images. In this phase, the loss function is dedicatedly designed based on the binary cross entropy (BCE) loss and the mean squared error (MSE) loss in order to maximize the performance of artifact elimination. These two phases work cooperatively to remove the wrap-around artifact while improving the image quality. Experiments, in terms of quantitative metrics and qualitative visualizations, demonstrate the high potential of the proposed method in the reduction of the wrap-around artifact.

The rest of this study is organized as follows. Section 2 details the proposed framework. Section 3 presents the experiments and detailed analysis regarding the wrap-around artifact removal. Finally, we conclude the work of this study in section 4.

## 2. Methodology

In section 2, we first propose a technique to simulate the wrap-around artifact on the MRI image. Then, a dataset is formed by pairing the artifact-free MRI image with the artifacted MRI image obtained from the proposed simulation technique. At last, the dataset is employed to train a deep learning network to implement the removal of the wrap-around artifact.

### 2.1. Artifact Simulation

For the MRI image with the wrap-around artifact, two factors affect the perceptual quality of the image, including the size and intensity of the wrapped area. Therefore, we generate the wrap-around artifact based on these two factors. Given an artifact-free MRI image ***I*** ∈ ℝ^*M*×*N*^, we produce the artifact layer ***Î*** by horizontally shifting the pixels in ***I*** as


(1) $$\hat{I}(\hat{m},\hat{n})=\{\begin{array}{ll} 0, & d+\hat{n}\leq N\\ I(\hat{m},d+\hat{n}-N)\cdot r, & \text{otherwise} \end{array}$$

where $\hat{I}\left(\hat{m},\hat{n}\right)$ indicates the pixel of ***Î*** located at $\left(\hat{m},\hat{n}\right)$, *d* ∈ [1, *N*] is the shift distance of ***I*** meaning that ***I*** is shifted horizontally by *d* columns, and *r* > 0 determines the intensity of the artifact layer. This study only considers the horizontal shift, implying that the wrap-around artifact only appears on either the right side or the left side of the image. However, it is straightforward to apply the proposed method to the situation of vertical shift.

After obtaining the artifact layer, the wrap-around artifact can be produced by directly adding the image and the artifact layer together. However, doing so will change the image contrast as the pixel value of the wrapped area is increased after the summation. Such a change will increase the difference between the light and dark areas of the image leading to that light areas become lighter and dark areas become darker. Consequently, the simulated artifact is inconsistent with the clinical practice. Herein, we propose a technique to circumvent these problems. Let ***F*** and $\hat{F}$ be the binary patterns of ***I*** and ***Î***, respectively, satisfying


$$F(m,n)=\{\begin{array}{ll} 1, & I(m,n)>0\\ 0, & \text{otherwise} \end{array};\;\hat{F}(m,n)=\{\begin{array}{ll} 1, & \hat{I}(m,n)>0\\ 0, & \text{otherwise} \end{array}.$$

We first overlay the image ***I*** with the artifact layer ***Î*** as


(2) $$\begin{array}{l} I_{r}=\left(I+\hat{I}\right)⊙F, \end{array}$$

where ⊙ indicates the element-wise multiplication. The summation of ***I*** and ***Î*** will lead to the artifact appearing on the blank area of the image. This kind of artifact information does not corrupt the image, and can be easily eliminated by applying the binary pattern of ***I***. Therefore, this study is only interested in the artifacts that contaminate the image information of ***I***. We apply ***F*** in Equation (2) to remove the artifact on the blank area of the image.

Then, the wrapped area of the image can be calculated by $V=\left(F+\hat{F}\right)⊙F$. The elements in ***V*** involves three different values, such as 0, 1, and 2. The pixels in the wrapped area are marked as 2. Therefore, we can obtain the location and size of the wrapped area by counting the number of elements of 2 in ***V***. Following this, we calculate the brightness ratio between the non-wrapped area and the wrapped area in the original artifact-free image. When artifacts are generated, we maintain the same ratio to avoid the problem of uneven brightness. Let *I*_1_ and *I*_2_ be the summation of the brightness in the unwrapped-area and wrapped-area of the image, written by


(3) $$\begin{array}{l} \begin{array}{cc} I_{1}=\sum I\left(m,n\right), & \text{for }V\left(m,n\right)=1\\ I_{2}=\sum I\left(m,n\right), & \text{for }V\left(m,n\right)=2. \end{array} \end{array}$$

The final wrapped-around artifact is generated as


(4) $$I_{s}(m,n)=\{\begin{array}{ll} I_{r}(m,n)\cdot \frac{I_{2}}{I_{1}}, & \;V(m,n)=2\\ I_{r}(m,n), & \text{otherwise}. \end{array}$$

**Figure 1:**
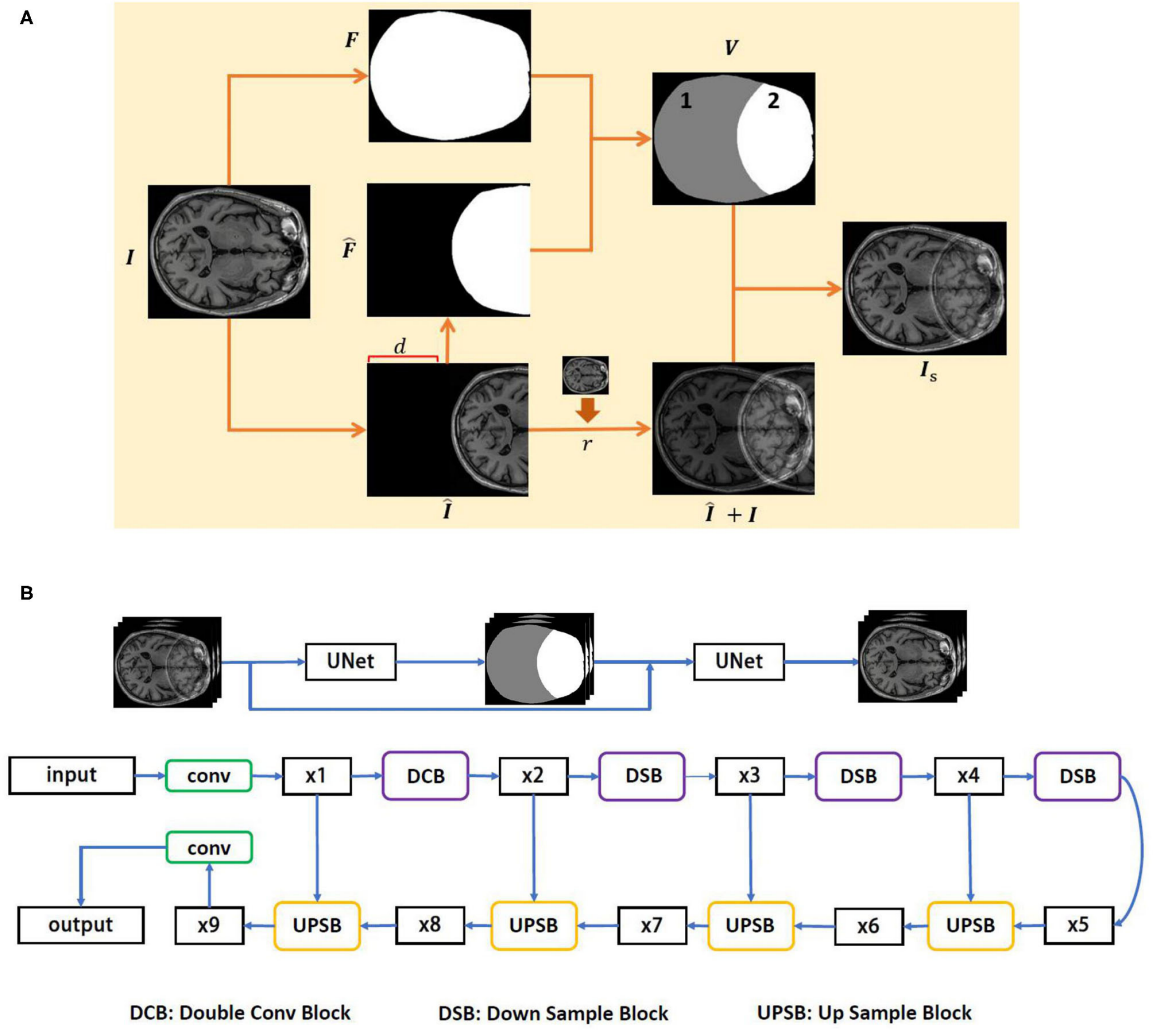
The proposed wrap-around artifact reduction framework. **(A)** The process of the wrap-around artifact simulation. **(B)** The architecture of the DUARN method.

The complete process of the wrap-around artifact simulation is presented in Figure 1A. The proposed simulation technique allows us to overcome the problem of data shortage. Hence, we can produce adequate artifact resources to facilitate the development of the artifact reduction technique. Toward this end, a deep learning-based method for the reduction of the wrap-around artifact is proposed.

### 2.2. Problem Formulation

In general, the observed image ***Y*** can be represented using a discrete linear model, written by


(5) $$\begin{array}{l} WI_{s}+\epsilon =Y\approx I, \end{array}$$

where ***I***_*s*_ and ***I*** are the artifacted and artifact-free images, respectively. ***W*** is a linear operator representing various operations against the image quality, i.e., the convolution operation in the K-space for image deblurring or the non-local means filtering for image denoising. ϵ is a bias term. Our purpose is to solve ***W*** in Equation (2), which is an ill-posed inverse problem that the solution of ***W*** is generally underdetermined. The priori knowledge of ***I***_*s*_, is therefore, required in order to constrain the solution space of ***W***. In other words, we hope to find ***W*** such that


(6) $$\begin{array}{l} L=\frac{1}{2}||I-Y|{|}_{2}^{2}+\lambda R\left(I_{s}\right) \end{array}$$

reaches minimum, where $\frac{1}{2}||I-Y|{|}_{2}^{2}$ is known as data term. The regularization term λ*R*(***I***_*s*_) with the regularization parameter λ is utilized to alleviate the problem of ill-posedness and *R*(***I***_*s*_) generally involves *l*_*q*_-norms. Equation (6) can be solved by learning-based method, such as the gradient descent method, to iteratively minimize the difference between ***I*** and ***Y*** to the local minimum.

### 2.3. Learning-Based Artifact Reduction

A deep learning-based method is herein proposed to solve Equation (6). The proposed method is constructed by two U-net networks; thus, we name it as Dual U-net Artifact Reduction Network (DUARN). The two U-nets correspond to two phases of DUARN, namely, artifact estimation and deep elimination. In the first phase, we train a U-net by pairing the artifacted MRI images with the binary artifact pattern aiming at accurately predicting the artifact area from the input artifacted image. The BCE loss is adopted in the network training owing to its powerful performance of binary image prediction. In the second phase, we rewrite Equation (6) as


(7) $$\begin{array}{l} L=aL_{MSE}⊙P_{1}+bL_{BCE} \end{array}$$

where ***P***_1_ is a binary pattern of the artifacted image obtained from the first phase of DUARN. When the pixel of ***P***_1_ is in the area of artifact, it is equal to 1 otherwise equal to 0. We use ***P***_1_ to maximize the learning efficiency of the second deep neural network. Since this study only deals with the wrap-around artifact, we assume that there is no other artifact existing outside the wrapped area of the image. Hence, when the network learns from the loss function, we set a relatively small weight for those losses outside the wrap-around artifact area, thereby improving the efficiency and accuracy of the network. $L_{MSE}=\frac{1}{N}\overset{N}{\underset{i=1}{\sum }}{\left(I_{i}-Y_{i}\right)}^{2}$ indicates the pixel-wise image domain MSE loss, where ***I***_*i*_ and ***Y*** are the *i*−th pixel value of ***I*** and ***Y***. $L_{BCE}=-\frac{1}{N}\overset{N}{\underset{i=1}{\sum }}Y_{i}\log\left(p\left(Y_{i}\right)\right)+\left(1-Y_{i}\right)*\log\left(1-p\left(Y_{i}\right)\right)$ refers to the BCE loss that minimizes the average probability error between the target and predicted images for each pixel. Herein, we adopt the BCE loss to penalize the misalignment of boundaries. *a* and *b* are small positive real numbers, satisfying *a* + *b* = 1. Empirically, we set *a* = 0.75 and *b* = 0.25.

The architecture of DUARN is illustrated in Figure 1B. The DUARN contains two U-nets, each of which involves four scales, such as 64, 128, 256, and 512. In the input layer, 64 filters with kernel size of 3 × 3 and ReLU as an activation function are applied. Following the input layers, there are four convolution layers (encoder) and four transposed convolution layers (decoder) with each followed by batch normalization and ReLU layers. The skip connection between the 2 × 2 strided convolution (downscaling) and 2 × 2 transposed convolution (upscaling) are employed in order to supplement the reconstruction details with different level of features. Finally, a 1 × 1 convolution layer is used to predict a single channel image as the output of the network.

## 3. Experiments and Analysis

**Figure 2:**
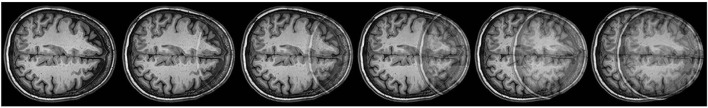
MRI images with different degrees of wrap-around artifact. From the left to right is the ground truth image, minor artifact, mild artifact, moderate artifact, severe artifact, and non-diagnostic.

In this section, we evaluate the proposed DUARN method with respect to its quantitative and qualitative performance. First, we enlist the help of radiologist to select 140 artifact-free and high perceptual quality MRI images (T1-weighted). The invited radiologist who has over 5 years of clinical experience in the brain radiology. Following the MRI data acquisition, we simulate the wrap-around artifact on the 140 artifact-free MRI images using the method proposed in section 2.1. We generate five different degrees of the wrap-around artifacts corresponding to five distortion levels of the image, in which the distortion level of 1, 2, 3, 4, and 5 indicate minor artifact, mild artifact, moderate artifact, severe artifact, and non-diagnostic as suggested by Liu et al. (2017, 2020a) and Liu and Li (2020). The simulation process of the artifact is carried out under the guidance of the radiologist who visually assesses the quality of each simulated image and recommends the parameter values of *d* and *r* in Equation (1) to ensure the generated image matches the desired distortion level. Examples of the simulated MRI images are presented in Figure 2 with the distortion level ranging from 1 to 5. The ground truth image is also provided on the left of Figure 2. It is observed that the wrap-around artifact on the minor artifacted MRI image is insignificant in terms of the area size and the intensity of the artifact. Images of such a quality may still be useful if the diagnostic area of interest is outside the artifact. Correspondingly, the MRI images with severe and non-diagnostic artifact can hardly be useful under any clinical situations.

We then produce the dataset of artifacted MRI images for training the proposed DUARN method. Since we have 140 artifact-free images, the produced dataset yields to a total number of 700 artifacted images. The dataset is split into two non-overlapped parts, i.e., training data and testing data with the standard ratio of 80/20%. We train the DUARN on the training data and test it on the testing data. We at first train the first U-net of DUARN, where the Adam optimizer is adopted with the initial learning rate of 0.0001, batch size of 2, and momentum of 0.8. When the training process is complete, we train the second U-net using the artifacted MRI image and the output of the first U-net as its inputs. The Adam optimizer is also applied to the second U-net with the initial learning rate of 0.00001, batch size of 1, and momentum of 0.9. The early stopping scheme is employed in the training process of both U-nets for the prevention of overfitting. In addition, the conventional data augmentation techniques, such as image flipping, rotating, and brightness adjustment, are adopted to boost the network performance.

**Figure 3:**
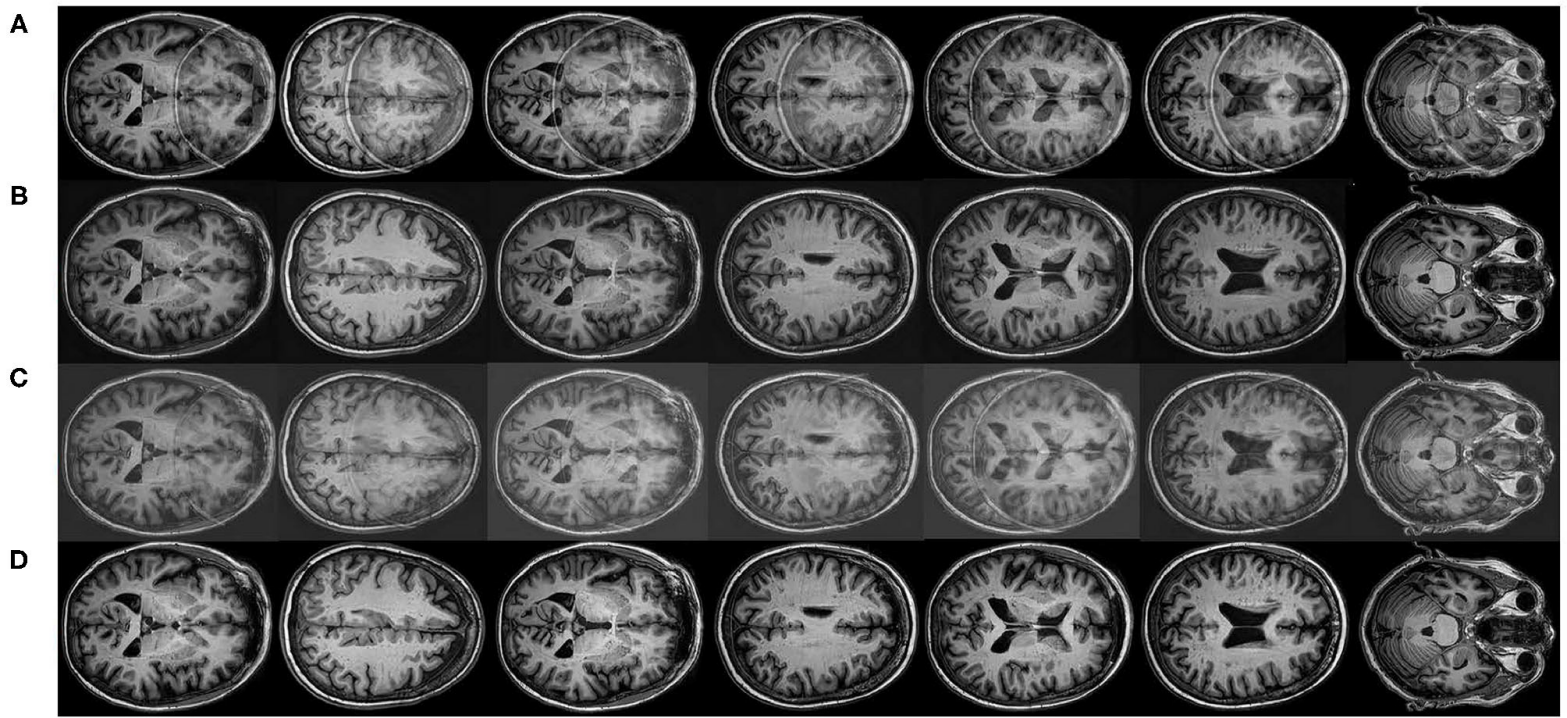
Qualitative visualizations. **(A)** Artifacted MRI images involving mild, moderate, severe, and non-diagnostic wrap-around artifacts. **(B)** Reconstructed MRI images from the DUARN method. **(C)** Reconstructed MRI images from the MARC method. **(D)** Ground truth images.

In order to vividly demonstrate the performance of the proposed method, we compare it quantitatively and qualitatively with the state-of-the-art artifact reduction method in Tamada et al. (2020). Tamada et al. (2020) proposed an artifact reduction method, namely motion artifact reduction based on convolutional neural network (MARC) method, to remove the motion ghost from the MRI images. In the MARC method, a convolutional neural network (CNN)-based network was trained to extract the artifact components from the artifacted images. The artifacts can be, therefore, removed by subtracting the extracted artifact component from the input image. The targeted artifact in Tamada et al. (2020) is similar to the wrap-around artifact since both of them belong to the aliasing of the image. We adapt the MARC method to implement the wrap-around artifact reduction and present the results of the MARC method and DUARN method in Figure 3. As can be observed, both methods are capable of eliminating the wrap-around artifact to a certain extent while the qualitative performance of the DUARN method is notably better than the MARC method, especially for those high distortion level image, i.e., 2nd and 5th images. In addition, we noticed that although the MARC method can alleviate the wrap-around artifacts, noise may be introduced into the images, resulting in further degradation of image quality. This is inconsistent with our purpose of obtaining high-quality artifact-free MRI image. On the contrary, the DUARN method can maintain the high perceptual quality after the artifact removal.

Finally, we evaluate the quantitative performance of the DUARN method by quantifying the quality of the reconstructed MRI image. Numerous image quality metrics have been proposed in the last decades each with their respective merits (Zhang et al., 2011; Mittal et al., 2012; Min et al., 2017, 2019, 2020c). In this study, we adopt two widely used metrics to measure the MRI image quality, including the peak signal-to-noise ratio (PSNR) and the structural similarity index measure (SSIM) (Wang et al., 2004). Table 1 tabulates the PSNR and SSIM from the DUARN and MRAC methods. The testing data contain 140 images with 28 images for each artifact type. We calculate the average PSNR and SSIM for each artifact type, and the overall in Table 1 refers to the average PSNR and SSIM for all the 140 testing images. As can be observed, the DUARN model achieves superior performance in the evaluation of all types of artifacts. More importantly, when the degree of image distortion increases, the performance of MARC method shows a clear downward trend. Comparatively, the DUARN method can still maintain a robust performance and even has a slight upward trend. This implies that the capability of the DUARN method will not be affected by the distortion level of the image. Such a feature is essential because clinical trials often face artifacted MRI images with various distortion levels, which may exceed the scope of the test samples. An artifact removal technique with stable performance can exert promising application value in practice. The DUARN method can be also combined with other image enhancement techniques, such as contrast stretching and histogram equalization, to further improve the perceptual quality of the reconstructed MRI image. This can be considered in the future work. In addition, since the DUARN method combines two losses of the BCE loss and the MSE loss in the network training, we are interested in the individual contribution of each loss in the performance of the proposed method. Toward this end, we introduce each loss to the network training of the DUARN method and quantify the performance of each loss by the PSNR and SSIM. The experimental results are presented in Table 1, where DUARN1 and DUARN2 indicate the DUARN method with the MSE loss and the BCE loss, respectively. As observed, the MSE loss brings more contributions in the DUARN method, and the combination of these two losses earns the best performance, which evidences that the BCE loss and the MSE loss play complementary roles in the DUARN method.

**Table 1:** The SSIM and PSNR from DUARN and MARC methods.

	**Minor artifact**	**Mild artifact**	**Moderate artifact**	**Severe artifact**	**Non-diagnostic**	**Overall**
**SSIM**						
MARC	0.9033	0.8949	0.9006	0.8766	0.8739	0.8899
DUARN1	0.9221	0.9292	0.9339	0.9384	0.9401	0.9327
DUARN2	0.8941	0.8901	0.8867	0.9012	0.8993	0.8943
DUARN	0.9536	0.95577	0.9594	0.9654	0.9684	**0.9605**
**PSNR**						
MARC	22.2786	20.7544	21.0742	17.6049	15.9149	19.5254
DUARN1	23.4502	23.6031	23.0125	24.5327	26.0182	24.1233
DUARN2	20.1963	19.7167	19.5369	22.3562	22.6943	20.9001
DUARN	24.3683	24.1942	24.0889	25.7176	27.6271	**25.1992**

*DUARN1 and DUARN2 indicate the DUARN method with the MSE loss and the BCE loss, respectively. Overall indicates the average values of SSIM and PSNR for all the 140 testing images. The highest performance values on each evaluation index are highlighted with boldface*.

## 4. Conclusion

This study deals with the wrap-around artifact of the MRI image, wherein two contributions are made. We first propose a simulation technique to generate the wrap-around artifact on the MRI image. The design of the proposed method is based on the image quality assessment scheme and with the assistance of an experienced radiologist, which allows the simulated artifact resources to match clinical situations. Then, we propose a novel artifact reduction technique, based on the deep neural network, to implement the elimination of the wrap-around artifact. This technique composes two U-net networks corresponding to two phases, such as artifact estimation and deep elimination. Dedicated losses are designed in order to maximize the effectiveness of artifact removal while improving the perceptual quality of the reconstructed MRI image. Extensive experiments are carried out to evaluate the quantitative and qualitative performance of the proposed method, with the results demonstrating the superiority of the proposed method against the state-of-the-art method.

## Data Availability Statement

The raw data supporting the conclusions of this article will be made available by the authors, without undue reservation.

## Author Contributions

RH, YL, and RY developed the theoretical formalism, performed the analytic calculations, and carried out the experiments. XL supervised the project. All authors contributed to the final version of the manuscript.

## Funding

This work was supported in part by the China Postdoctoral Science Foundation under Grant 2019M650686.

## Conflict of Interest

The authors declare that the research was conducted in the absence of any commercial or financial relationships that could be construed as a potential conflict of interest.

## Publisher's Note

All claims expressed in this article are solely those of the authors and do not necessarily represent those of their affiliated organizations, or those of the publisher, the editors and the reviewers. Any product that may be evaluated in this article, or claim that may be made by its manufacturer, is not guaranteed or endorsed by the publisher.
